# Influence of Vitamin D on Periodontal Inflammation: A Review

**DOI:** 10.3390/pathogens12091180

**Published:** 2023-09-20

**Authors:** Mohamed M. Meghil, Christopher W. Cutler

**Affiliations:** Department of Periodontics, The Dental College of Georgia, Augusta University, Augusta, GA 30912, USA

**Keywords:** gingivitis, periodontitis, immunology, vitamin D

## Abstract

The active form of vitamin D is the hormonally active 1,25(OH)_2_D_3_ (Vit D) vitamin, which plays an important role in bone biology and host immunity. The vitamin D receptor (VDR) is a nuclear ligand-dependent transcription factor expressed by many cells. Ligation of VDR by VitD regulates a wide plethora of genes and physiologic functions through the formation of the complex Vit D-VDR signaling cascade. The influence of Vit D-VDR signaling in host immune response to microbial infection has been of interest to many researchers. This is particularly important in oral health and diseases, as oral mucosa is exposed to a complex microbiota, with certain species capable of causing disruption to immune homeostasis. In this review, we focus on the immune modulatory roles of Vit D in the bone degenerative oral disease, periodontitis.

## 1. Introduction

### 1.1. Periodontitis and the Host Inflammatory Response

Periodontitis affects nearly half of the population over age 30 in the US [1]. The periodontium, consisting of gingiva, periodontal ligament and alveolar bone, provides the tooth-supporting apparatus. Periodontitis lesions are thought to start as gingivitis lesions, wherein the inflammation is confined to the gingival tissue without destruction of the underlying soft and hard tissue. Gingivitis is also characterized by the presence of intact clinical attachment. Clinical attachment loss occurs when the gingival and periodontal fibers are destroyed. As a consequence, the integrity of the junctional epithelium becomes disrupted, and the gingival pocket epithelium migrate apically towards the root apex, resulting in deepening of the periodontal pocket. Even though there are histopathological similarities between gingivitis and periodontal disease, it is still unknown how gingivitis progresses to periodontal disease [2,3]. Previously, periodontitis was classified into chronic and aggressive periodontitis, according to the old classification system of periodontal disease [4]. Currently, according to the consensus report of the 2017 World Workshop on the Classification of Periodontal and Peri-Implant Diseases and Conditions, there is no evidence that supports the distinction between chronic and aggressive periodontitis. Current periodontitis classification is based on stages that are defined by severity (level of interdental clinical attachment loss, radiographic bone loss and tooth loss) and grades that demonstrate the biologic characteristics of the disease (evidence of rapid progression, risk factors, anticipated response to therapy, and effects on systemic health) [5]. Given the high complexity and the broad aspects of the PD inflammatory response, this review will focus on the influence of Vit D on a specific immune event, that is, the activation of immature DCs through their pathogen-associated molecular patterns receptors, and the subsequent antigen presentation to naïve CD4+ T cells which leads to different Th phenotypes.

The periodontal environment provides a rich niche for commensal and pathogenic oral microbes to live and grow within. The periodontal microenvironment in periodontal health is characterized by a balanced immuno-inflammatory state that is capable of maintaining host–microbe homeostasis. In addition, the microbial composition of dental plaque biofilm is in a state of balance and stability, comprising mainly symbiotic biofilm of Gram-positive aerobic bacteria. However, the progression from gingivitis to periodontitis is associated with a dramatic shift in the microbial community structure, mainly comprising dysbiotic and anaerobic microbial biofilm [6]. Some of the dysbiotic microbial biofilm implicated in the development of severe forms of periodontal disease include (*Porphyromonas gingivalis* (*P. gingivalis*), *Treponema denticola* (*T. denticola*) and *Tannerella forsythia* (*T. forsythia*)) [7]. Furthermore, in periodontitis, the immunoregulatory mechanisms are disrupted, leading to dysregulation of the host immune response. Periodontitis starts with the accumulation of periodontal pathogen in the dental plaque. The innate immune system drives the initial host response to the bacteria of the plaque biofilm as a first line of defense through its basic mechanisms: anatomical and physical barriers, secretory molecules, and cellular components such as PMNs, macrophages and dendritic cells (DCs). Tissue residents, Langerhans cells (LCs), also play an immune-modulatory role in periodontitis. In an experimental model of periodontitis in mice, it has been reported that mucosal LCs induced differentiation of *P. gingivalis*-specific Th17 [8].

The pathogenesis of periodontitis (PD) has been long attributed to excessive gingival influx of polymorphonuclear leukocytes (PMNs). PMNs encounter oral biofilm, release MMPs, IL-6 and other cytokines that promote bone loss [9,10,11]. Over the years, studies of PD lesion have indicated the involvement of other immune cells, including tissue resident dendritic cells, B cells, plasma cells, M1 macrophages [12,13] and CD4+ T cells [14]. Particular attention is drawn to DCs, which bridge the innate and adaptive immunity, and direct T cell effector responses. Immature DCs (iDCs) infiltrate gingival tissues and other peripheral sites in PD [15,16], capturing subgingival oral microbes such as *P. gingivalis* [15,16] and *Fusobacterium nucleatum* [17], which can regulate DC maturation and migration to secondary lymphoid organ (SLO) [15,18,19,20]. Persistent local inflammatory signals promote unrestrained activation of DC maturation in peripheral tissues in situ, as in Crohn’s disease [21] arthritis [22] and periodontitis [14,23,24,25]. Intensive immune clusters of matured CD83+ DCs with CD4+ T cells are found in lamina propria of PD patients [14,23,24,25], evocative of ectopic lymphoid foci [26,27]. DCs can shape Th17/Treg balance towards Th17 to eradicate invading bacteria, but Th17/Treg imbalance promotes osteoclast-mediated alveolar bone loss [28,29]. Immune-regulatory DCs can also induce FoxP3+ Tregs, which inhibit Th17 responses and attenuate experimental PD [30,31].

DCs are professional antigen-presenting cells (APCs) that play an important role in both innate and adaptive immune responses [32]. Unlike other leukocytes, DCs lack the classic leukocyte lineage markers (e.g., CD3, CD14, CD15, CD19, CD20, and CD56), while expressing high levels of major histocompatibility complex (MHC) class II molecules [33]. DCs are generally divided into plasmacytoid (CD11c^−^CD123^+^) and myeloid (CD11c^+^CD123^−^) subpopulations [34,35,36]. Myeloid DCs upregulate the expression level of the costimulatory molecules CD83, CD208 and CCR7 upon maturation, and are involved in cellular immunity against intracellular pathogens. On the other hand, plasmacytoid DCs produce large amounts of type-I interferon; hence, it is believed to play a crucial role in host immunity against viral infection [37].

Since the discovery of DCs in the 1970s [38,39], they were considered as immune-stimulatory cells until their tolerogenic function was observed in the 1990s [40,41]. Immature DCs capture antigens in the peripheral tissues, phagocytose them, and then process them for antigen presentation to naïve T cells in MHC-II molecules. Upon pathogen recognition, DCs are exposed to pro-inflammatory signals that lead to their maturation and the upregulation of their expression of MHC-II, co-stimulatory (e.g., CD40, CD80, CD86, and CD83) lymph node-homing migratory chemokine receptors, and the inflammatory cytokines such as IL-12. At the same time, they down-regulate phagocytic mediators such as C-type lectins. After that, DCs migrate to regional lymph nodes, where they activate effector T-cell type (e.g., Th1, Th17) responses via presentation of their processed antigen peptides to T-cells [42]. DCs have been reported in human studies to infiltrate the oral lamina propria in PD with CD83+ matured DCs [43]. These mature DCs form immune complexes in situ with CD4+ T cells, also called oral lymphoid foci [14,43]. These foci are similar to ectopic lymphoid follicles found in other chronic inflammatory diseases [44], and are thought to result from continuous exposure to oral microbes and repeated damage to the oral mucosal epithelium [45,46,47]. In the experimental PD model in mice, a destructive role for in situ-matured DCs in promoting Th17-mediated alveolar bone loss has been documented [48]. DCs also can maintain the immature status when exposed to internal antigens (e.g., apoptotic cells) in the absence of strong pro-inflammatory stimulants, resulting in expressing low levels of MHC-II, co-stimulatory molecules and cytokines, inducing T-cell anergy [49]. On the other hand, using a ligature PD model in mice, recent studies have reported that Tregs may become phenotypically unstable and lose their anti-osteoclastogenic phenotype [50]. In addition, DCs exposed to TGF-β1 and IL-10 possess impaired maturation, and promote regulatory T-cell (Tregs) responses, while inhibiting Th17 effectors. Furthermore, cytokines loaded into DC-derived exosomes have been shown to inhibit bone loss in experimental PD in mice [48].

In their immature state, DCs patrol tissues and blood circulation. They are capable of capturing different pathogens through the recognition of pathogen-associated molecular patterns (PAMPs), such as lipopolysaccharide (LPS), fimbriae or flagellin [51]. Immature DCs identify microbes in the peripheral tissues via a set of extracellular and intracellular pattern recognition receptors (PRRs). Upon encountering a pathogen, PRRs trigger a proinflammatory maturation program by activating intracellular signal transduction pathways, orchestrating the activation of gene expression and the production of proteins that are crucial for shaping the innate and the adaptive host immune responses [51]. PRRs can be classified into Toll-like receptors (TLRs), C-type lectin receptors (CLRs), nucleotide-binding domain, leucine-rich repeat (LRR)-containing (or NOD-like) receptors (NLRs), RIG-I-like receptors (RLRs), and AIM2-like receptors (ALRs). These superfamilies of receptors can be subdivided into two main groups: membrane-bound receptors and unbound intracellular receptors. The membrane-bound group includes the TLRs and CLRs, and are found at the cell surface or on endosomal compartments. TLRs and CLRs recognize microbial ligands in the extracellular environment and within endosomes. On the other hand, the unbound intracellular receptors include NLRs, RLRs, and ALRs, and are located in the cytoplasm, where they capture intracellular pathogens [52].

Expansion of blood myeloid CD1c^+^(BDCA-1) CD209^+^ DCs is reported in peripheral blood of periodontitis patients; moreover, this expansion further increases 24 h after mechanical debridement (scaling and root planning), which provokes a bacteremia [15]. In addition, it has been shown that periodontitis patients with existing coronary artery disease have a further increase in blood myeloid DCs. These DCs circulate in the blood stream carrying microbial cargo, including *P. gingivalis* and other periodontal pathogens. Moreover, postmortem analysis of coronary artery samples from periodontitis patients who had coronary artery disease shows co-localization of CD209 (DC-SIGN), myeloid DCs marker, with *P. gingivalis* minor fimbria protein (mfa-1) in the atherosclerotic plaques [15].

Fimbriae are adhesins expressed by periodontal pathogens, such as *P. gingivalis*, and constitute a potent virulence factor [53]. Fimbriae bind to other bacteria in the periodontal microenvironment, facilitating colonization of the oral mucosa and also the invasion of host cells [54,55]. *P. gingivalis* has two distinct types of fimbriae, long (major) and short (minor) fimbriae [56,57], both of which are involved in colonization and adhesion activities [58]. The role of *P. gingivalis*’s fimbria in the invasion of DCs is notable. While the minor fimbria targets the C-type lectin DC-SIGN on DCs for invasion and survival within [15,57,59], the major fimbriae target TLR1/2 on DCs [60]. *P. gingivalis* minor fimbria ‘dial down’ the signals received from the major fimbriae, inhibiting apoptosis and autophagy of *P. gingivalis*-loaded DCs [61]. The exploitation of DC migratory functions by periodontal pathogens could be responsible for the microbial dissemination from peripheral sites to systemic circulation. In the periodontium, DCs interact with and activate T-cells in the lamina propria at the oral lymphoid foci [14].

It is worth repeating that PMNs are the most dominant leukocytes recruited to the periodontal tissue in periodontitis [62]. Studies have reported that individuals with congenital deficiencies in PMN function or numbers are more prone to severe forms of periodontitis, and hence, this suggests that PMNs play a major role in periodontal tissue homeostasis [63,64]. PMNs, when poorly regulated, undergo a process of activation and subsequent lysis, which leads to the destruction of the periodontium. Even though the initial response to periodontal pathogens involves recruitment of PMNs, the initiation and regulation of the adaptive immune response is principally mediated by DCs under the influence of the proper cytokine microenvironment [65]. DCs are crucial for priming CD4 naïve T cells to differentiate into Th1, Th2, Th17, follicular helper T (Tfh) and T regulatory cells (Treg). On the other hand, each T-cell subset has specific immune homeostatic function, and is responsible for production of cytokines that shape the nature of the host immune response. Th1 cells are mainly involved in cellular immunity and produce interleukin (IL)-2 and interferon gamma (IFN-γ) [66]. Th2 cells mediate the humoral response to infection by the production of IL-4, IL-5, and IL-13, which, in turn, play a role in the production of pathogen-specific antibodies via the activation of B cells [67]. Th17 cells can affect the periodontal environment by increasing PMN recruitment to the diseased site [68]. On the other hand, Treg cells down-regulate T-cell response and reduce the pro-inflammatory host response [30]. Ultimately, Th17 cells promote alveolar bone loss via activating an excessive pro-inflammatory response and tipping the OPG/RANKL ratio, while Treg cells results in the reduction in alveolar bone loss [69].

### 1.2. Vitamin D and the Immune Modulatory Effects

Vitamin D (Vit D) are a group of fat-soluble hormones that play a well-known role in bone development via increasing the absorption of calcium. Vitamin D3 (cholecalciferol) is generated in the skin through photochemical transformation of 7-derydrocholesterol after exposure to ultraviolet light, while Vitamin D2 (Ergocalciferol) is present in some foods and dietary supplements [70]. Even though the structural difference in these two forms does not affect the biological activity of their active metabolites, it alters their metabolism and binding to the carrier protein Vit D binding protein (DBP), a protein that transports Vit D metabolites in the blood [71]. It is noteworthy that less than 1% of Vit D circulates in the free form, whereas around 85–90% circulates bound to DBP, and 10–15% bound to albumin [72,73]. Both forms of Vit D are metabolized to 25OHD_3_ in the liver, and then undergo hydroxylated in the kidneys by CYP27B1 to produce the active hormone involved in calcium absorption in the gut, 1,25(OH)_2_D_3_ (calcitriol) [74,75]. In addition, 1,25(OH)_2_D has also been shown to be produced by different subsets of human monocyte-derived macrophages, and that Vit D metabolism is a macrophage polarization-dependent process [76]. Vit D deficiency is defined as having a 25-hydroxyvitamin D level equal to or less than 20 ng/mL, and insufficiency is a concentration of 21 to 29 ng/mL [77]. Although it is difficult for many to attain adequate vit D levels from diet alone, most people achieve sufficient amounts via skin exposure to sunlight [78].

Studies have shed light on the multi-faceted immune regulatory roles of Vit D, and its profound anti-inflammatory effects [79]. Vit D deficiency, for example, increases the incidence and severity of chronic inflammation in patients with periodontitis [80], cardiovascular disease [81,82], inflammatory bowel disease [83], asthma [84], chronic obstructive pulmonary disease (COPD) [85], and autoimmune diseases [86]. Vit D supplementation in those deficient patients has been shown to reduce the severity of chronic inflammatory diseases and levels of pro-inflammatory mediators [86,87].

At the cellular level, Vit D exerts its actions via binding to its receptor, Vit D receptor (VDR), a member of the nuclear receptor superfamily which are ligand-activated transcription regulator molecules [88]. Vit D shapes the host immune response during the onset of inflammation and infection. Following vaccination, the pro-inflammatory cytokine responses of immune cells that express VDR, such as monocytes, macrophages, DCs, and T-cells, are moderated by Vit D [89,90]. Inflammasomes are a group of large multiprotein complexes assembled around several innate immune proteins in response to recognition of pathogens, leading to the direct activation of caspase-1, which subsequently induces cleavage and unconventional secretion of active IL-1β and IL-18 [91]. Notably, the reduction in IL-1β induced by Vit D through the inhibitory effect of VDR on NALP3-inflammasome activation [92] results in the alleviation of PD [93]. Furthermore, low Vit D levels are associated with increased incidence of autoimmune diseases such as rheumatoid arthritis (RA), multiple sclerosis (MS), and systemic lupus erythematosus (SLE) [94]. The influence of Vit D and VDR on T-cell function, differentiation, and homeostasis has been reported in several studies. In VDR-knockout mice (VDR-KO), VDR has been shown to be non-essential for the development of CD4+, CD8+, and CD4+ FOXP3+ T-cells, though its absence contributes to the development of autoimmune diseases [95,96,97].

**Figure 1:**
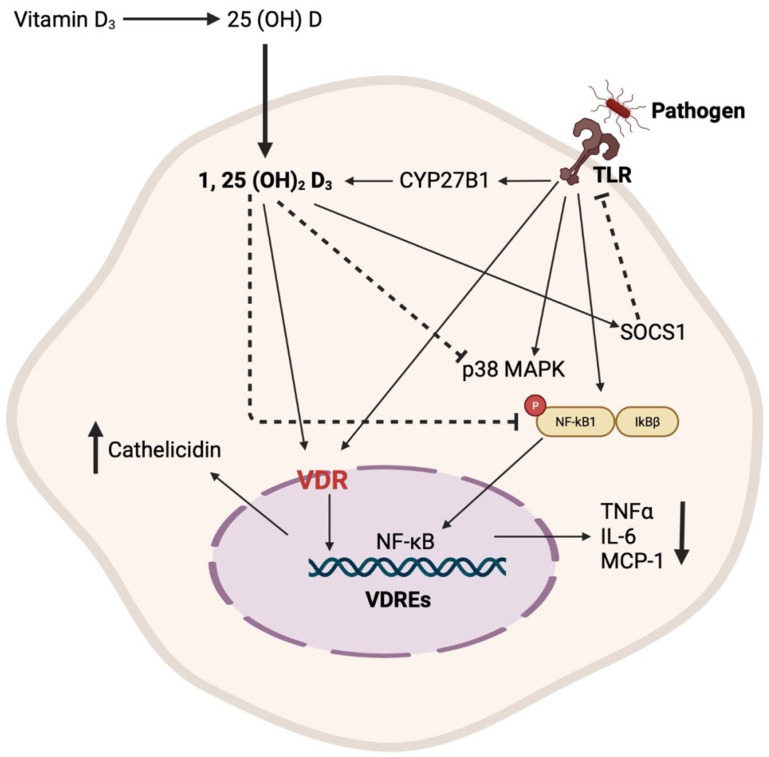
Schematic representation of the primary mechanisms through which vitamin D regulates the innate immune response.

VDR expression plays a crucial role in inducing antimicrobial innate immune response to certain pathogens. The expression levels of VDR and CYP27B1 upregulate as a result of the activation of TLR1/2 receptor, leading to the production of cathelicidin. In turn, the increase in CYP27B1 results in the increased production of 1,25(OH)_2_D_3_, further leading to the activation of VDR, resulting in the increased transcription of target genes located in the regulatory regions of 1,25(OH)_2_D_3_ via vitamin D response elements (VDREs) [98,99,100]. Furthermore, 1,25(OH)_2_D_3_ regulates the TLR signaling pathway by stimulating SOCS1, providing a negative regulatory mechanism to the innate immune response [101]. In addition, both forms of vitamin D, 1,25(OH)_2_D_3_ and 25(OH)D_3,_ have been shown to inhibit LPS-induced p38, IL-6, and TNFα production [102]. Moreover, 1,25(OH)_2_D_3_ can inhibit the activation of NF-κB, resulting in a reduction in the expression of MCP-1 and IL-6 [103], thereby leading to decreased recruitment of monocytes/macrophages and overall inflammation (Figure 1).

**Figure 2:**
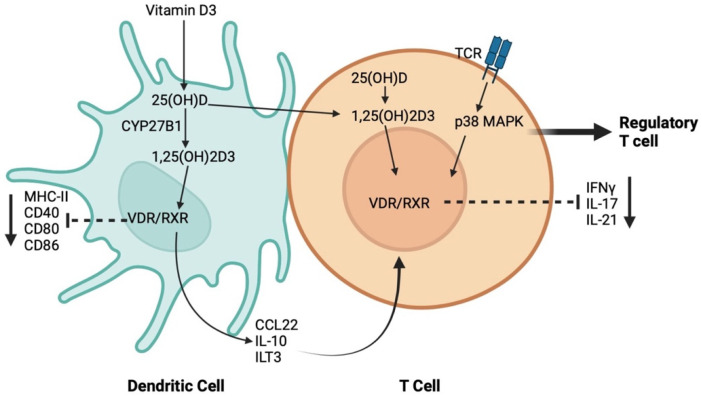
Schematic representation of the mechanisms through which vitamin D regulates the crosstalk between dendritic cells (DCs) and T-cell.

DCs are the most potent antigen-presenting cells for CD4 naïve T-cells due to their high expression of major histocompatibility complex-II (MHC-II) and co-stimulatory molecules. Several studies have unraveled the influence of 1,25(OH)_2_D_3_ on the homeostatic functions of DCs by shaping their tolerogenic characteristics, inhibiting the differentiation, maturation, and immunostimulatory capacity of human DC, in a VDR-dependent manner [104,105]. In addition, 1,25(OH)_2_D_3_ induces tolerogenic properties of DCs via decreasing the surface expression of MHC-II, and costimulatory and maturation markers such as CD40, CD80, and CD86, upregulating inhibitory immunoglobulin-like transcript 3 molecules, and enhancing the secretion of chemokine (C–C motif) ligand 22 and IL-10 [104,106]. The induction of DC tolerogenic phenotype results in the suppression of the inflammatory response of T-cells through inducing a regulatory T-cell response [106]. Studies have shown that the relationship between Vit D and T-cell response is bi-directional. 1,25(OH)_2_D_3_ has been reported to only inhibit the production of proinflammatory cytokines, including IFNγ, IL-17, and IL-21 in CD4^+^ CD25^−^ T lymphocytes, but it also promotes the development of CD4^+^ FOXP3^+^ regulatory T-cells [107]. On the other hand, T-cell responses can differentially control Vit D metabolism. While IFNγ of the T-helper (Th)-1 effector response results in the upregulation of CYP27B1, leading to the enhanced bioconversion of 25(OH)D_3_ to 1,25(OH)_2_D_3_, IL-4 production in the Th-2 response results in the catabolism of 25(OH)D_3_ to the inactive metabolite 24,25(OH)_2_D_3_ [108]. Interestingly, naïve T cells do not express VDR, but VDR expression is induced by TCR signaling via the activation of the alternative MAP kinase p38 pathway [109]. Although the results of these studies suggest a potential role for Vit D on both the innate and adaptive host immune response, the underlying mechanism by which Vit D partakes in this process is still unknown (Figure 2).

### 1.3. The Influence of Vit D on the Immune Response in the Periodontium

Vit D plays several essential roles in the periodontium, including modulating proinflammatory cytokine production, stimulating the secretion of antimicrobial peptides, and activating hydrogen peroxide release in monocytes [100,110,111]. The innate immune response against periodontal pathogens includes secretion of antimicrobial peptides. Antimicrobial peptides including β-defensins and cathelicidin are crucial for the naturalization of bacterial endotoxins and lipopolysaccharides [112,113]. The interaction between pathogen-associated molecular patterns (PAMPs) and TLR1/2 of monocytes, macrophages and keratinocytes in the periodontium has been shown to induce the expression of VDR and the production of the active form of Vit D, 1,25(OH)_2_D by these cells. Subsequently, the signal transduction of the 1,25(OH)_2_D/VDR axis induces the expression of genes encoding the β-defensins and cathelicidin [114,115,116], which provides protection against bacterial biofilm that is crucial for the development of bacterial plaque-induced periodontal disease [117].

Alveolar bone loss in periodontitis is preceded by osteoclast activation or osteoclastogenesis. This is induced by the receptor activator of nuclear factor kappa-Β ligand (RANKL) binding to RANKL receptor on osteoclasts, thereby activating them [118]. IL-17 cytokine production by Th-17 T-cells increases in periodontal disease. In turn, Th17-derived IL-17 and TNF-*α* directly or indirectly promote the expression of RANKL. In addition, LPS release by anaerobic periodontal pathogens stimulates TLRs signaling, which upregulates the expression of RANKL by host cells such as fibroblasts, osteoblasts, T- cells and B-cells, thereby increasing the differentiation and activation of osteoclasts. IL-17 can also upregulate the expression of RANKL by osteoblasts and CD4+ T cells. PMNs, macrophages, and Th1 cells also can directly promote osteoclastogenesis by secretion of TNF-*α*, which together contribute to periodontal alveolar bone resorption [119]. Calcitriol is the biologically active hormone responsible for regulating the systemic calcium and phosphate homeostasis. Hence, it is not only required for the mineralization of cartilage and bone matrix, but it also plays a crucial role in the regulation of gene expression and differentiation [120]. Interestingly, a recent study in rats showed that Vit D deficiency negatively affects the levels of serum RANKL and RANKL/OPG ratio [121]. Cyp27B1 plays a role in the production of calcitriol form vitamin D 25OHD_3_. Animal studies have reported that deletion of the Cyp27B1 gene results in increased alveolar bone loss and increased production of pro-inflammatory cytokines, including interleukin-1β (IL1-β), tumor necrosis factor-α (TNF-*α*), and matrix metalloproteinases 3 and 6 (MMP-3 and MMP-8) [122]. In addition, a study has highlighted the role of calcitriol in the regulation of periodontal health using ligature-induced periodontitis in CYP27B1 knockout mice [123]. While CYP27B1 knockout mice show increased alveolar bone loss and gingival inflammation relative to control mice, exogenous supplementation of calcitriol reverses the effect of CYP27B1 gene deletion and restores alveolar bone loss and gingival inflammation [123]. In addition to decreasing alveolar bone loss, calcitriol intervention increases the anti-inflammatory cytokines IL-4 and IL-10 in LPS-induced periodontitis in rats [124]. Moreover, Vit D has been shown to reduce alveolar bone loss and modulate the AhR/NF-κB/NLRP3 inflammasome pathway in mice infected by *P. gingivalis* [93]. Comparing 10 weeks to 12 months 1α(OH)ase^-/-^ mice and wild-type littermates, Gong et al. have shown increased bone loss, NF-κB p65 and CD3 positive cells, gene expression levels of IL-1β, TNF-α, MMP-3 and -8, and decreased number of osteoblasts in 1α(OH)ase^−/−^ group compared with the wild type in an age-dependent manner [122].

Vit D exerts a potent antimicrobial effect against periodontal pathogens by directly inhibiting the growth of the bacteria [125] and LPS-induced inflammation [126] or facilitating the production of antimicrobial peptides such as β-defensins and cathelicidins [100,111,127]. Calthelicidin LL-37 has a potent antimicrobial activity against both Gram-positive and Gram-negative bacteria [128,129]. An in vitro study by Yang et al. on keratinocytes showed that LL-37 promotes autophagy in keratinocytes, and reduces the number of live *P. gingivalis* [130]. Calthelicidin affects multiple aspects of the innate host immune response, including chemotaxis, cytokines production, vascular permeability and neutralization of bacterial endotoxins [131]. Furthermore, calcitriol upregulates cathelicidin hCAP-18 gene expression [100]. Another in vitro study on epithelial cells has shown that calcitriol also significantly increases the production of calthelicidin LL-37 mRNA, CD14 expression, and triggers receptor expressed on myeloid cells-1 (TREM-1) in human gingival epithelial cells, suggesting that calcitriol supplementation enhances the innate immune response in the periodontium [112]. In addition, a recent study on human gingival fibroblasts has shown that Vit D exerts an anti-inflammaging effect and reduces oxidative stress through the activation of Nrf2 signaling pathway [132].

Clinically, cross-sectional studies have reported that normal Vit D serum levels is associated with stable periodontium, which might confer some resistance to periodontal disease progression. The positive role of Vit D in maintaining calcium and bone homeostasis must be noted, which in turn can increase the mineral density of alveolar bone, and thus may reduce alveolar bone resorption [74,133,134,135]. Furthermore, the negative role of 1,25 dihydroxyvitamin D/VDR signaling pathway on the transcription of genes encoding pro-inflammatory cytokines can also suppress other signaling pathways responsible for cyclo-oxygenase-2 (COX-2) and prostaglandin pathways, and can inhibit the production of matrix metalloproteinases (MMPs) that cause destruction of soft and hard tissues in the periodontium [74,135,136].

Several other studies reported that sufficient Vit D levels may improve periodontal health [133,137,138,139], while other reports provide conflicting results on the association of periodontal health with Vit D levels [140,141]. Cross-sectional analyses of the National Health and Nutrition Examination Survey (NHANES) database have shown that Vit D serum levels are negatively associated with periodontal disease and severe clinical attachment loss [142,143]. In addition, it has been shown that not only the increased Vit D levels reduces gingival inflammation [144], but also, the anti-inflammatory effect of Vit D is dose-dependent [145]. In addition, prospective and observational studies have demonstrated that serum Vit D level is negatively associated with tooth loss [135,146].

Vit D regulates autophagy through different signaling pathways [147]. It induces autophagy activation as an antimicrobial defense mechanism. Vit D transcriptionally promotes the expression of the autophagy protein ATG16, and low levels of Vit D results in decreased expression of ATG16 [148]. In addition, Vit D increases the expression of cathelicidin to promote the expression of Beclin1 and ATG5, resulting in autophagy activation [149]. Our previous clinical study of periodontitis patients demonstrated decreased expression levels of the autophagy-related proteins ATG5-12 conjugates, ATG16L1 and ATG7, at the protein and mRNA levels [80]. Interestingly, Vit D supplementation rescued autophagy in periodontitis patients, and increased the expression of Beclin1, ATG5-12 conjugate, ATG16L1 and ATG7 [80].

## 2. Conclusions

**Figure 3:**
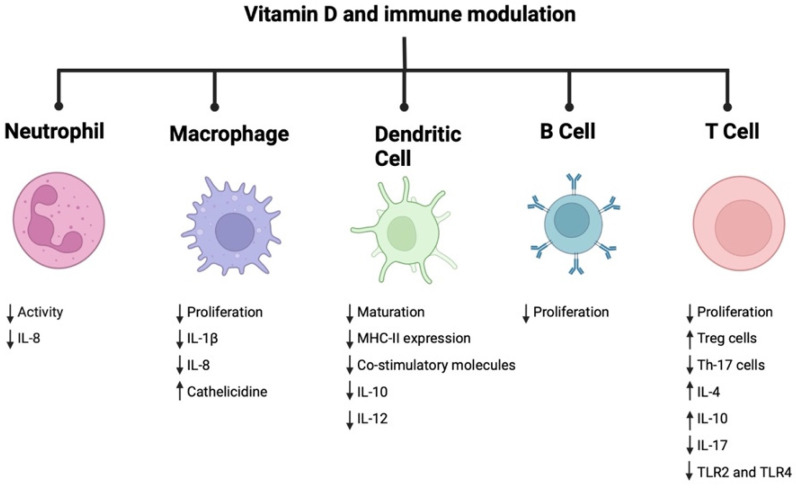
Immune modulatory role of vitamin D.

Vit D is an essential vitamin for overall human health. In addition to its direct effect on bone metabolism, it has a regulatory role in both the innate and adaptive arms of the immune system (Figure 3). Its essential function in curbing inflammation responsible for chronic debilitating diseases such as periodontitis is notable. To better understand its influence on clinical symptoms and the rate of alveolar bone loss in periodontitis, randomized controlled trials in Vit D-deficient patients are needed.

## Data Availability

No new data were created.
